# Age-Associated YBX1 Phosphorylation Regulates the Keratinocyte Senescence-Associated Secretory Phenotype Through Translational Control

**DOI:** 10.3390/cells15171586

**Published:** 2026-09-01

**Authors:** Valdi Ven Japranata, Michelle Liu, Fabiana Boncimino, Emery Di Cicco, Sara Palumbo, Kristina Todorova, Enkhtuul Gantumur, Stefano Sol, Anna Mandinova

**Affiliations:** 1 Cutaneous Biology Research Center, Massachusetts General Hospital and Harvard Medical School, Boston, MA 02129, USA; 2 Broad Institute of Harvard and MIT, 7 Cambridge Center, Cambridge, MA 02142, USA; 3 Harvard Stem Cell Institute, 7 Divinity Avenue, Cambridge, MA 02138, USA

**Keywords:** skin aging, keratinocyte senescence, YBX1, phosphorylation, post-transcriptional regulation

## Abstract

Skin aging is characterized by epidermal atrophy, reduced keratinocyte proliferation, and the accumulation of senescent cells that sustain a chronic, low-grade inflammatory secretome known as the senescence-associated secretory phenotype (SASP). We previously showed that Y-box binding protein 1 (YBX1) limits keratinocyte senescence in human epidermis by acting as a translational repressor of SASP chemokines, including CXCL1 and IL8. How this brake is regulated during skin aging, however, remains undefined. Here we demonstrate that, although total YBX1 protein is reduced in keratinocytes from aged human epidermis, the fraction of phosphorylated YBX1 (pYBX1) is increased relative to young donors, resulting in an elevated pYBX1/total YBX1 ratio that correlates with chronological age. Because pYBX1 is predominantly nuclear, whereas unphosphorylated YBX1 is cytoplasmic, this shift is predicted to deplete the cytoplasmic pool available for translational repression of CXCL1 and IL8. Consistent with this model, treatment of immortalized (Ker-CT) and primary human keratinocytes (HK) with the PI3K inhibitors PI-103 and GDC-0941 suppressed YBX1 phosphorylation without altering total YBX1 abundance, thus retaining YBX1 in the cytoplasm. This allows cytoplasmic YBX1 to re-engage and repress the translation of *CXCL1* and *IL8* mRNAs, lower chemokine secretion, and reduce the fraction of senescent keratinocytes. Moreover, conditioned medium from PI-103- or GDC-0941-treated cells sufficiently decreased senescence in recipient cells, consistent with a paracrine, secretome-mediated effect. Together, these findings support an association between YBX1 phosphorylation and SASP chemokine output in aging human keratinocytes and nominate YBX1-directed modulation as a candidate strategy for selectively attenuating senescence-associated secretory phenotypes in intrinsic skin aging.

## 1. Introduction

Aging is a complex biological process characterized by a progressive decline in cellular and tissue integrity and function [1]. In skin, aging manifests as impaired barrier integrity, reduced regenerative capacity, loss of elasticity, and increased susceptibility to disease [2]. These changes result from the combined influence of extrinsic factors, such as ultraviolet radiation and environmental exposures, and intrinsic mechanisms driven by genetic mutations, cellular senescence, and molecular regulatory pathways that disrupt epidermal homeostasis over time [2,3]. Although extrinsic contributors to skin aging have been extensively investigated, intrinsic aging programs remain comparatively understudied despite their central role in determining baseline aging trajectories and interindividual variability. This gap limits the development of interventions targeting the underlying biology of skin aging, as current approaches largely address the downstream manifestations. Therefore, identifying intrinsic molecular regulators of age-associated skin dysfunction is an important unmet need.

Cellular senescence is a key intrinsic driver of skin aging, contributing to epidermal thinning, alterations in metabolism and gene expression, and pro-inflammatory signaling that affects tissue homeostasis [4]. Senescent keratinocytes undergo stable cell-cycle arrest accompanied by extensive transcriptional remodeling and acquisition of a senescence-associated secretory phenotype (SASP), a pro-inflammatory secretome that influences the surrounding microenvironment [5]. Despite its importance, the upstream regulators coordinating SASP-associated gene expression in keratinocytes remain incompletely defined.

Among the RNA-binding proteins implicated in the regulation of SASP, Y-box binding protein 1 (YBX1) has been demonstrated to suppress cellular senescence in human keratinocytes by repressing the translation of cytokine mRNAs, including *CXCL1* and *IL8* [6]. Y-box binding protein 1 (YBX1) is a multifunctional DNA- and RNA-binding protein containing a conserved cold shock domain that regulates gene expression through phosphorylation-dependent subcellular localization [7]. Cytoplasmic, unphosphorylated YBX1 regulates mRNA stability and translation, while phosphorylation promotes nuclear translocation and transcriptional activity [7,8]. Phosphorylated YBX1 (pYBX1), particularly at Ser102 residue, has been studied in cancer, including squamous cell carcinoma, where it promotes proliferation and stress-response pathways [9]. However, whether this phosphorylation switch is involved during physiological skin aging remains unexplored.

Because phosphorylation determines YBX1 localization and regulatory function, alterations in YBX1 phosphorylation may differentially influence SASP gene expression in aging keratinocytes. We hypothesized that inhibition of YBX1 phosphorylation may retain YBX1 in the cytoplasm to suppress SASP gene expression via post-transcriptional regulation. Here, we investigated the phosphorylation-dependent dynamics of YBX1 in regulating SASP in human keratinocytes, aiming to define an intrinsic molecular mechanism contributing to skin aging.

## 2. Methods

### 2.1. Cell Culture

Human abdominal skin samples were obtained from donors undergoing elective plastic surgery. Donors were stratified into three age groups: young (≤40 years), middle-aged (41–59 years), and old (≥60 years). Skin samples were processed immediately after collection for keratinocyte isolation. Tissue samples were incubated in dispase solution (StemCell Technologies, Vancouver, BC, Canada, #07913) at 4 °C overnight to separate the epidermis from the underlying dermis. The epidermis was then mechanically minced into small pieces and subjected to enzymatic dissociation with 0.05% Trypsin-EDTA 1× (Gibco, Grand Island, NY, USA, #25300-054) for 10 min at 37 °C to generate a single-cell suspension of keratinocytes. The primary human keratinocytes were then passed through a 70 µm cell strainer, seeded in a precoated dish (StemCell Technologies, #352350), and cultured in Keratinocyte SFM (K-SFM; Gibco, #10724-011) supplemented with 0.3 ng/mL human recombinant epidermal growth factor (EGF; Gibco, #10450-013), 30 μg/mL bovine pituitary extract (BPE; Gibco, #13028-014), and 1% antibiotic/antimycotic (Gibco, #15240-062). Cultures were maintained at 37 °C in 5% CO_2_. Cells at 60–70% confluence were split 1:3 using TrypLE™ Select (Thermo Fisher Scientific, Waltham, MA, USA, #12604-013). All donor samples were screened and confirmed negative for hepatitis B virus, hepatitis C virus, HTLV-1, HTLV-2, HIV-1, and HIV-2.

Ker-CT cells (ATCC^®^, Manassas, VA, USA, CRL-4048™) and OKF6-TERT2 cells (a gift from James Rheinwald, Brigham and Women’s Hospital, Boston, MA, USA) were cultured in K-SFM (Gibco, #10724-011) supplemented with 0.3 ng/mL human recombinant EGF (Gibco, #10450-013), 30 μg/mL BPE (Gibco, #13028-014), and 1% antibiotic/antimycotic (Gibco, #15240-062).

Cell cultures were routinely tested for *Mycoplasma* with a Mycoplasma PCR Detection Kit (Abcam, Cambridge, UK, #ab289834) every two weeks, and contaminated cultures were discarded.

### 2.2. MTT Assay

Ker-CT cells or primary human keratinocytes were seeded at 10,000 cells/well in 96-well plates. After 24 h, cells were treated with DMSO (Sigma-Aldrich, St. Louis, MO, USA, #D4540), PI-103 (Selleckchem, Houston, TX, USA, #S1038), GDC-0941 (Selleckchem, #S1065), SL0101 (MedChemExpress, Monmouth Junction, NJ, USA, #HY-15237), or MK-2206 (MedChemExpress, #HY-10358) at the indicated concentrations for 48 h, following which a standard MTT assay was performed with Triazolyl blue tetrazolium bromide (MTT), a membrane-permeable dye (Abcam, #ab146345). Cells were incubated for 3 h at 37 °C in the dark in a medium containing 0.5 mg/mL MTT solution. The medium was subsequently removed and replaced with isopropanol, followed by a further 30-min incubation in the dark at 37 °C. Absorbance was then measured at 570 nm and 620 nm using an EnVision 2104 Multilabel Reader (PerkinElmer, Shelton, CT, USA), and the 620 nm reading was subtracted from the 570 nm reading to correct for background interference.

### 2.3. Luciferase Assay

The 3′UTR luciferase reporter constructs (for 3′-UTR_CXCL1, 3′-UTR_IL8-M2, and empty vector) were generated based on sequences described in a previously published study [6]. Ker-CT cells were transiently transfected with these constructs using Lipofectamine™ 2000 transfection reagent (Thermo Fisher Scientific, #11668027). At 48 h post-transfection, Ker-CT cells were treated with DMSO (Sigma-Aldrich, #D4540), PI-103 (Selleckchem, #S1038), or GDC-0941 (Selleckchem, #S1065). After 2 h of drug treatment, luciferase activity was measured using a Luciferase Reporter Assay Kit (Promega, Madison, WI, USA, #E1910), according to the manufacturer’s recommendations, and luminescence was determined using an EnVision 2104 Multilabel Reader (PerkinElmer).

### 2.4. SDS-PAGE and Western Blotting

Cells were lysed in RIPA buffer (Boston BioProducts, Milton, MA, USA, #BP-115). Samples were prepared after mixing three parts of the clear lysates with one part of 4× NuPAGE™ LDS Sample Buffer (Invitrogen, Carlsbad, CA, USA, #NP008) and β-mercaptoethanol (EMD Millipore Corp., Burlington, MA, USA, #444203). Samples were heated at 95 °C for 5 min and loaded onto denaturing SDS-PAGE gels (NuPAGE™ Bis–Tris 4–12% Mini Protein Gels, Thermo Fisher Scientific, #NP0335BOX, #NP0322BOX, #NP0336BOX, #NP0329BOX), followed by Western blotting. Membranes were blocked with 5% non-fat dry milk (Lab Scientific bioKEMIX, Danvers, MA, USA, #M0841) in 1× Tris-buffered saline containing 0.05% Tween-20 (TBST; Boston BioProducts, #IBB-180) for 1 h at room temperature, followed by incubation with primary antibodies overnight at 4 °C. The following primary antibodies were used: anti-phospho-YBX1 (Ser102) (C34A2) (1:1000, rabbit; Cell Signaling Technology, Danvers, MA, USA, #2900), anti-YBX1 (1:2500, rabbit; Abcam, #ab76149), anti-vinculin (1:10,000, mouse; Santa Cruz Biotechnology, Dallas, TX, USA, #sc73614), anti-AKT (1:1000, rabbit; Cell Signaling Technology, #9272), anti-pAKT (1:1000, rabbit; Cell Signaling Technology, #4058), anti-p16^INK4a^ (1:1000, rabbit; Abcam, #ab108349), anti-p21^CIP1^ (1:1000, rabbit; Cell Signaling Technology, #2947), anti-phospho-p90^RSK^ (Ser380) (D3H11) (1:1000, rabbit; Cell Signaling Technology, #11989), anti-p90^RSK^ (1:1000, rabbit; Cell Signaling Technology, #9355), anti-phospho-S6 ribosomal protein (Ser235/236) (D57.2.2E) (1:2000, rabbit; Cell Signaling Technology, #4858), and anti-S6 ribosomal protein (5G10) (1:500, rabbit; Cell Signaling Technology, #2217) diluted in 5% bovine serum albumin (BSA; Sigma-Aldrich, #A7030) in TBST (Lab Scientific bioKEMIX, #M0841). After three 5-min washes with TBST, membranes were incubated with horseradish peroxidase (HRP)-conjugated Goat anti-Rabbit IgG (H + L) (1:10,000; Invitrogen, #31460) and Goat anti-Mouse IgG (H + L) (1:10,000; Invitrogen, #31430) secondary antibodies for 1 h at room temperature. Protein bands were visualized using Supersignal™ West Pico PLUS Chemiluminescent Substrate (Thermoscientific, Rockford, IL, USA, #34580) and detected by autoradiography. Band intensities were quantified using ImageJ v1.54t for Mac by calculating the area under the curve for each band.

### 2.5. Translating Ribosome Affinity Purification (TRAP)

TRAP was performed to isolate ribosome-RNA complexes. For each condition, 4 × 10^6^ cells were washed in ice-cold DPBS (Gibco, #14190-144) containing 10 µg/mL cycloheximide. Cells were lysed in 1 mL of lysis buffer (20 mM HEPES-NaOH pH 7.5, 2.5 mM MgCl_2_, 150 mM NaCl, 1% (*v*/*v*) Triton X-100, 0.5 mM DTT, protease/phosphatase inhibitor cocktail, RNase inhibitor, and 100 µg/mL cycloheximide) and rotated at 4 °C for 30 min. Whole-cell lysates were clarified by centrifugation at 18,000× *g* for 10 min at 4 °C. Lysates were pre-cleared with Dynabeads (Thermo Fisher Scientific, #2820407) for 1 h at 4 °C. In parallel, 4 µg of anti-9D5 antibody (Medical & Biological Laboratories, Tokyo, Japan, #RN004M) or mouse IgG control (ProteinTech, Rosemont, IL, USA, #65208-1-IG) was conjugated to 28 µL of Dynabeads M-280 sheep anti-mouse IgG, and the antibody-coupled beads were incubated with pre-cleared lysates for 1 h at 4 °C. Beads were then washed three times with a wash buffer (20 mM HEPES-NaOH pH 7.5, 2.5 mM MgCl_2_, 150 mM NaCl, 0.05% (*v*/*v*) Tween 20). Ten percent of the beads were reserved for protein analysis (resuspended in a 2× SDS sample buffer and heated at 65 °C, 10 min). For RNA recovery, the remaining beads were mixed with TRIzol^®^ reagent (Thermo Fisher Scientific), and RNA was purified with Direct-zol™ RNA Miniprep Kit (Zymo Research, Irvine, CA, USA, #R2052), including on-column DNase treatment. Purified RNA was subjected to reverse transcription using Maxima First Strand cDNA Synthesis Kit (Thermo Fisher Scientific, #FERK1671). Quantitative PCR was subsequently carried out using PowerUp™ SYBR™ Green Master Mix (Applied Biosystems, Woburn, MA, USA, #A25742) on a QuantStudioTM 3 (Applied Biosystems) or QuantStudio™ 5 (Applied Biosystems). Expression levels of target genes were normalized to the housekeeping gene *RPLP0* and expressed relative to the vehicle-treated control group (DMSO). Enrichment was calculated relative to input and normalized to the IgG control. Primer sequences used in this study are listed in Table 1.

### 2.6. Immunofluorescence Staining of Cultured Human Keratinocytes

Primary human keratinocytes were grown on 18 mm round glass coverslips (Fisherbrand, Ottawa, ON, Canada, #18CIR.-1) placed in 12-well culture plates until cells reached 60–70% confluency. Cells were then fixed with 4% paraformaldehyde (PFA; Boston BioProducts, #BM-155) for 10 min, followed by permeabilization with 0.1% Triton X-100 (Sigma-Aldrich, #T8787-250) in 1× PBS (Teknova, Hollister, CA, USA, #P1225) for 5 min. To prevent nonspecific antibody binding, cells were incubated in 2% BSA (Sigma-Aldrich, #A7030) in 1× PBS (Teknova, #P1225) for 1 h at room temperature.

Coverslips were subsequently incubated with primary antibodies overnight at 4 °C in a humidified chamber, followed by incubation with fluorescent secondary antibodies for 1 h at room temperature. Primary antibodies used were anti-YBX1 (1:100, rabbit; Abcam, #ab76149) or anti-phospho-YBX1 (Ser102) (C34A2) (1:100, rabbit; Cell Signaling Technology, #2900). Secondary labeling was performed using Alexa Fluor^TM^ 488-conjugated anti-rabbit IgG (1:1000; Invitrogen, #A21206), together with Alexa FluorTM Plus 647 Phalloidin (1:200; Invitrogen, #A30107) to visualize the actin cytoskeleton.

Following each incubation step, coverslips were washed twice with 1× DPBS (Gibco, #14190-144) for 5 min per wash. Nuclei were counterstained with Hoechst 33342 (Life Technologies, Carlsbad, CA, USA, #H3570), and coverslips were mounted using VECTASHIELD^®^ Antifade Mounting Medium (Vector Laboratories, Newark, CA, USA, #H-1000-10).

Fluorescent images were acquired using an Axio Observer Z1 microscope (ZEISS, Oberkochen, Germany) and processed using Zen Microscopy 3.11 software (ZEISS). Fluorescence intensity was quantified using QuPath v0.7.0 for Mac. Both image acquisition and subsequent analysis were performed by investigators blinded to experimental conditions to minimize observer bias.

### 2.7. Histology and Immunofluorescence Staining of Human Skin

Human skin tissue samples were embedded in Tissue-Tek^®^ O.C.T. compound (Sakura Finetek, Torrance, CA, USA, #4583) within cryomolds and rapidly frozen to generate O.C.T. blocks for long-term storage at −80 °C. Frozen sections were prepared from these O.C.T.-embedded tissues using a cryostat (Leica Biosystems, Nußloch, Germany) at a thickness of 7 µm. Sections were allowed to air dry for 15 min prior to fixation with 4% PFA (Boston BioProducts, #BM-155) for 15 min. Following fixation, tissue sections were permeabilized using 0.1% Triton X-100 (Sigma-Aldrich, #T8787) in 1× PBS (Teknova, #P1225) for 30 min. Non-specific antibody binding was blocked by incubating sections in 5% normal donkey serum (SouthernBiotech, Birmingham, AL, USA, #0030-01) or 5% normal goat serum (Abcam, AB7481) diluted in 1× PBS (Teknova, #P1225) for 1 h at room temperature. Sections were then incubated with primary antibodies overnight at 4 °C in a humidified chamber, followed by incubation with fluorophore-conjugated secondary antibodies for 1 h at room temperature. Primary antibodies included anti-YBX1 (1:100, rabbit; Abcam, #ab76149), anti-phospho-YBX1 (Ser102) (C34A2) (1:200, rabbit; Cell Signaling Technology, #2900), anti Ki-67 (1:50, rabbit; Abcam, #ab16667), and anti-Keratin K5 (1:200, guinea pig; American Research Products, Waltham, MA, USA, #03-GP-CK5). Secondary antibodies consisted of Alexa Fluor^TM^ 488 anti-rabbit IgG (1:2000; Invitrogen, #A21206), Alexa Fluor^TM^ 488 anti-guinea pig IgG (1:1000; Invitrogen, #A11073), and Alexa Fluor^TM^ 568 anti-rabbit IgG (1:500; Invitrogen, #A10042). Nuclei were stained with 1 μg/mL Hoechst 33342 (Life Technologies, #H3570) and slides were mounted with VECTASHIELD^®^ Antifade Mounting Media (Vector Laboratories, #H-1000-10). Fluorescence imaging was conducted using an Axio Observer Z1 microscope (ZEISS), and the images were processed using Zen Microscopy 3.11 software (ZEISS). All samples within an experiment were imaged using identical acquisition settings to ensure comparability. Quantitative analysis of fluorescence signals was performed using ImageJ v1.54t for Mac. Image acquisition and downstream analysis were performed by investigators blinded to donor age group and experimental conditions to reduce observer bias.

### 2.8. Hematoxylin & Eosin (H&E) Staining of Human Skin

Frozen skin tissue sections were allowed to air dry for 15 min before fixation with 4% PFA (Boston BioProducts, #BM-155) for 15 min. Slides were washed briefly with 1× PBS (Teknova, #P1225), then incubated in Hematoxylin 2 (Epredia, #7231) for 45 s, followed by a 1-s dip in Eosin Y Alcoholic (Epredia, Portsmouth, NH, USA, #6766008). After each staining step, slides were rinsed in distilled water until the runoff ran clear. Tissue was dehydrated through a graded ethanol series: 70% for 2 min, 95% for 2 min, and 100% for 4 min. Slides were then cleared in xylene (Sigma-Aldrich, #534056) for 5 min before being mounted with VectaMount^®^ Express Mounting Medium (Vector Laboratories, #H-5700-60) and coverslipped with microscope cover glasses (Fisherbrand, #12541042). Histological morphology was visualized using a NanoZoomer S60 (Hamamatsu Photonics, Hamamatsu City, Japan), and all images were captured using identical acquisition settings to ensure comparability across samples. Epidermal thickness and rete ridge index were quantified in ImageJ v1.54t for Mac by investigators blinded to donor age to minimize observer bias.

### 2.9. Enzyme-Linked Immunosorbent Assay (ELISA)

Following treatment, cellular supernatants were harvested and centrifuged at 10,000× *g* for 10 min at 4 °C. Supernatant levels of CXCL1 and IL8 were quantified using the human CXCL1 DuoSet™ ELISA Development Systems (R&D Systems, Minneapolis, MN, USA, #DY275-05) and human IL8 DuoSet™ ELISA Development Systems (R&D Systems, #DY208-05), according to the manufacturer’s protocol. Absorbance was measured at 450 nm using an EnVision 2104 Multilabel Reader (PerkinElmer), and concentrations were calculated from a standard curve generated using the standards provided in each kit. Secreted CXCL1 and IL8 concentrations were normalized to cell number in the corresponding well to control for differences in cell density.

### 2.10. Senescence-Associated β-Galactosidase (SA-β-Gal) Staining

Growth media were removed from keratinocyte cultures, and cells were stained for senescence-associated β-galactosidase (SA-β-gal) activity using the Senescence β-Galactosidase Staining Kit (Cell Signaling Technology, #9860S) according to the manufacturer’s recommendations. Plates were incubated overnight at 37 °C in a dry incubator without CO_2_ to allow color development. Stained cells were subsequently examined using an Incucyte^®^ S3 Live Cell Analysis System (Sartorius, Göttingen, Germany) at 20× total magnification, acquiring both phase and near-infrared (NIR) channel images (NIR exposure time: 400 ms). The NIR channel signal, corresponding to the SA-β-gal blue precipitate, was pseudo-colored and overlaid onto the phase image. Senescent (SA-β-gal^+^) cells were identified by the presence of blue staining and quantified as a proportion of total cells within the same merged image.

### 2.11. Conditioned Medium Transfer Assay

Primary human keratinocytes were treated with DMSO (Sigma-Aldrich, #D4540), PI-103 (Selleckchem, #S1038), or GDC-0941 (Selleckchem, #S1065) at 1 µM for 24 h. To remove residual inhibitor, cells were then washed three times with 1× DPBS (Gibco, #14190-144) and refed with fresh, drug-free K-SFM. Cells were subsequently conditioned in this drug-free medium for 24 h, after which the conditioned culture medium was collected from each condition and used to treat a separate population of human primary human keratinocytes that had not been previously exposed to the drugs. Recipient cells were incubated with the conditioned medium for 48 h and then subjected to an SA-β-gal assay, performed according to the manufacturer’s instructions, to assess senescence induction.

### 2.12. siRNAs and Transfection Methods

Predesigned siRNAs targeting human YBX1 (Invitrogen, Cat. No. 4390824, IDs: s9732 and s9733) and control siRNAs (Invitrogen, AM4641) were used. Primary human keratinocytes at 60–70% confluence in 6-well plates were transiently transfected using HiPerFect Transfection Reagent (Qiagen, Venlo, The Netherlands). After 4 h incubation, the transfection medium was replaced with fresh K-SFM.

### 2.13. Statistical Analysis

All datasets derive from at least three independent experiments unless otherwise indicated. Data are presented as the mean of independent experiments ± SEM, as indicated. All statistical analyses were performed using GraphPad Prism software (version 10.0). For experiments comparing two groups, a paired or unpaired Welch’s two-tailed *t*-test was performed, whereas for experiments comparing more than two groups, one-way analysis of variance (ANOVA) was performed, as described in the figure legends.

## 3. Results

### 3.1. pYBX1/Total YBX1 Ratio Is Elevated in Old Human Epidermis

To investigate YBX1 in the context of skin aging, we assembled a cohort of human skin specimens from young (≤40 years) and old (≥60 years) donors. We first assessed that these tissues displayed the expected hallmarks of chronological aging. Immunofluorescence for K5 (basal keratinocytes marker) and Ki-67 (active proliferation marker) revealed a significant reduction in the proportion of Ki-67^+^ cells within the K5^+^ compartment in old relative to young skin (Supplementary Materials Figure S1A,B). Histological analysis further showed reduced epidermal thickness (Supplementary Materials Figure S1C,D) and a lower rete ridge index (Supplementary Materials Figure S1E), consistent with epidermal atrophy and flattening of the dermal-epidermal junction. Having verified that our cohort recapitulates the decline in keratinocyte proliferative capacity and epidermal architecture expected with age, we next analyzed YBX1 and pYBX1 in the same tissues.

**Figure 1:**
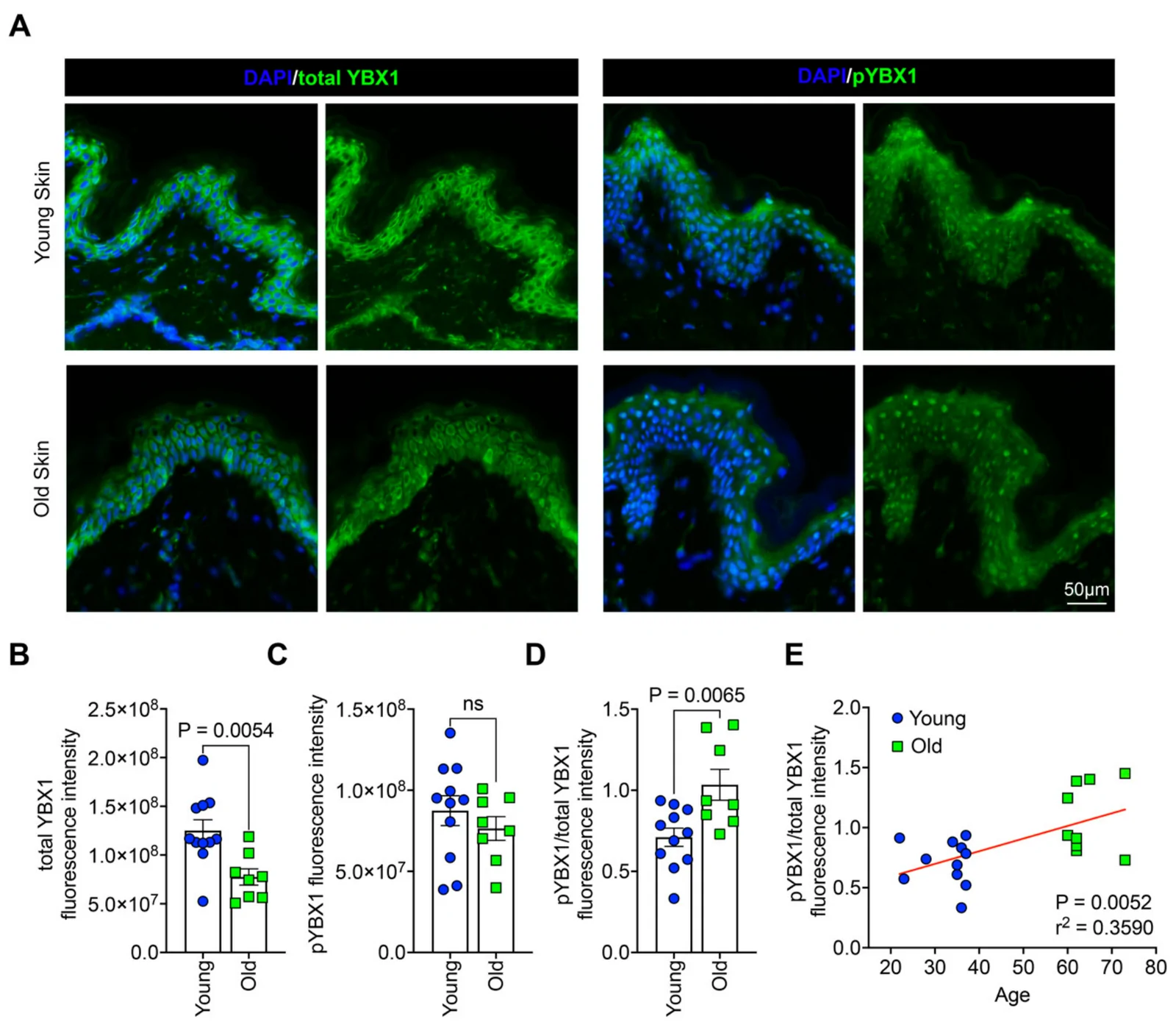
The pYBX1/total YBX1 ratio is elevated in old human epidermis despite reduced total YBX1. (**A**) Representative immunofluorescence images of YBX1 (**left panel**, green), pYBX1 (**right panel**, green), and DAPI (blue) in young (**upper panel**) and old (**lower panel**) human skin samples. Scale bar, 50 μm. (**B**,**C**) Quantification of total YBX1 (**B**) and pYBX1 (**C**) fluorescence intensity, and the pYBX1/total YBX1 (**D**) fluorescence intensity ratio in young and old human skin samples. ns = not significant. (**E**) Correlation between the pYBX1/total YBX1 fluorescence intensity ratio and donor chronological age. n = 11 young donors and n = 8 old donors. All values are shown as mean ± SEM; unpaired two-tailed *t*-test was performed for (**B**–**D**). Linear regression was performed for (**E**).

Immunofluorescence analysis showed that total YBX1 was mainly cytoplasmic, whereas pYBX1 was predominantly nuclear across all age groups (Figure 1A), consistent with the established notion that phosphorylation promotes YBX1 nuclear translocation [8]. Total YBX1 fluorescence intensity was significantly lower in old skin donors (Figure 1A,B), while pYBX1 intensity did not differ significantly between groups (Figure 1A,C). As a consequence, the pYBX1/total YBX1 ratio, a measure of the relative phosphorylation state of the protein, was significantly elevated in old skin (Figure 1D) and showed a significant positive association with donor chronological age across the sampled range (Figure 1E). These findings indicate that physiological skin aging is accompanied by a shift in the phosphorylation status of YBX1 despite an overall decline in total protein abundance.

### 3.2. PI3K/AKT Pathway Inhibition Suppresses YBX1 Phosphorylation and the Translation of CXCL1 and IL8 3′UTR Reporters

**Figure 2:**
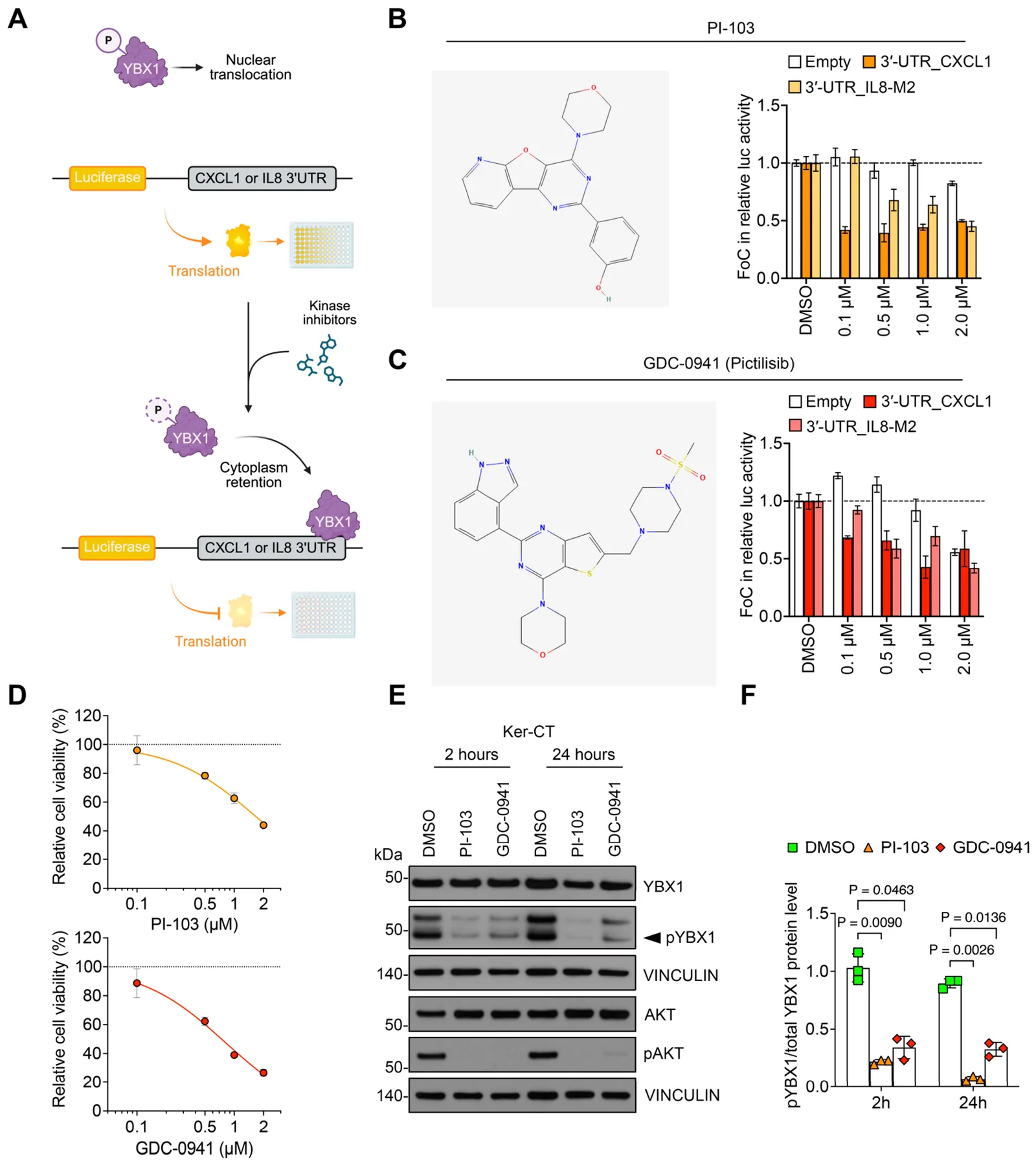
PI3K/AKT pathway inhibition suppresses YBX1 phosphorylation and translation of the *CXCL1* and *IL8* 3′UTRs reporters. (**A**) Schematic of the luciferase reporter assay used to screen for inhibitors of YBX1 phosphorylation in Ker-CT cells. (**B**,**C**) Chemical structure of PI-103 ((**B**), **left panel**) and GDC-0941 ((**C**), **left panel**). Luciferase reporter assay in Ker-CT cells transfected with 3′UTR-CXCL1, 3′UTR-IL8-M2, or empty vector; 48 h after transfection, cells were treated with the indicated concentrations of DMSO, PI-103 ((**B**), **right panel**), or GDC-0941 ((**C**), **right panel**). Relative luciferase activity was normalized to the DMSO treatment. (**D**) MTT assay in Ker-CT after 48 h of treatment with the indicated concentrations of DMSO, PI-103 (**upper panel**), or GDC-0941 (**lower panel**). Relative cell viability was normalized to DMSO. n = 3 biological replicates. (**E**) Western blot analysis of YBX1, pYBX1, AKT, and pAKT in Ker-CT cells treated with DMSO, 1 μM PI-103, or 1 μM GDC-0941 for 2 or 24 h. Vinculin was used as a loading control. (**F**) Quantification of pYBX1/total YBX1 protein levels from (**E**). n = 3 biological replicates. All values are shown as mean ± SEM; one-way ANOVA followed by Dunnett’s multiple comparisons test for (**F**). The schematic in (**A**) was created with BioRender.

Given the age-associated increase in pYBX1/total YBX1 ratio, we next sought to investigate the upstream signaling pathway responsible for this molecular change. Because of the established role of AKT in directing the nuclear translocation of YBX1 via phosphorylation of Ser102 [10], we examined whether pharmacologic inhibition of the PI3K/AKT pathway could modulate YBX1 phosphorylation and, consequently, its subcellular localization in keratinocytes. To test this hypothesis, Ker-CT, an immortalized human keratinocyte cell line, were transiently transfected with luciferase reporter constructs containing the 3′ UTRs of *CXCL1* or *IL8*, two established YBX1 translational targets [6]. In this system, increased cytoplasmic YBX1 represses reporter translation through binding to the 3′ UTR, resulting in reduced luciferase activity. We therefore tested two PI3K/AKT inhibitors, named PI-103 and GDC-0941, for their ability to promote YBX1-mediated translational repression (Figure 2A). Treatment with PI-103 and GDC-0941 resulted in a marked, dose-dependent reduction in luciferase activity, whereas the empty reporter was largely unaffected (Figure 2B,C).

To determine whether these effects were caused by cytotoxicity, Ker-CT viability was assessed following inhibitor treatment. The reduction in reporter activity occurred despite only modest, dose-dependent effects on Ker-CT viability over the same range of concentration (Figure 2D). As 1 µM produced a clear reduction in the CXCL1 and IL8 reporters without significantly affecting the empty control, we used this concentration for all subsequent experiments. Immunoblotting analysis of Ker-CT treated with PI-103 or GDC-0941 for 2 and 24 h confirmed that the reduction in pYBX1 band intensity was maintained at both time points, while there were no alterations in total YBX1 levels. In parallel, levels of phosphorylated AKT (pAKT), the active form of AKT, were reduced following treatment with PI-103 and GDC-0941, confirming effective inhibition of PI3K/AKT signaling (Figure 2E,F). Additionally, to confirm that these effects were not specific to a single cell line, we treated a second immortalized keratinocyte cell line, OKF6-TERT2, with PI-103 or GDC-0941 and observed a similar reduction in both pYBX1 and pAKT, with preserved total YBX1 levels (Supplementary Materials Figure S2A).

Together, these findings identify PI-103 and GDC-0941 as PI3K/AKT pathway inhibitors that suppress YBX1 phosphorylation and enhance YBX1-dependent post-transcriptional repression of *CXCL1* and *IL8*.

### 3.3. PI3K/AKT Pathway Inhibition Reduces YBX1 Phosphorylation Across Independent Human Keratinocyte Donors

**Figure 3:**
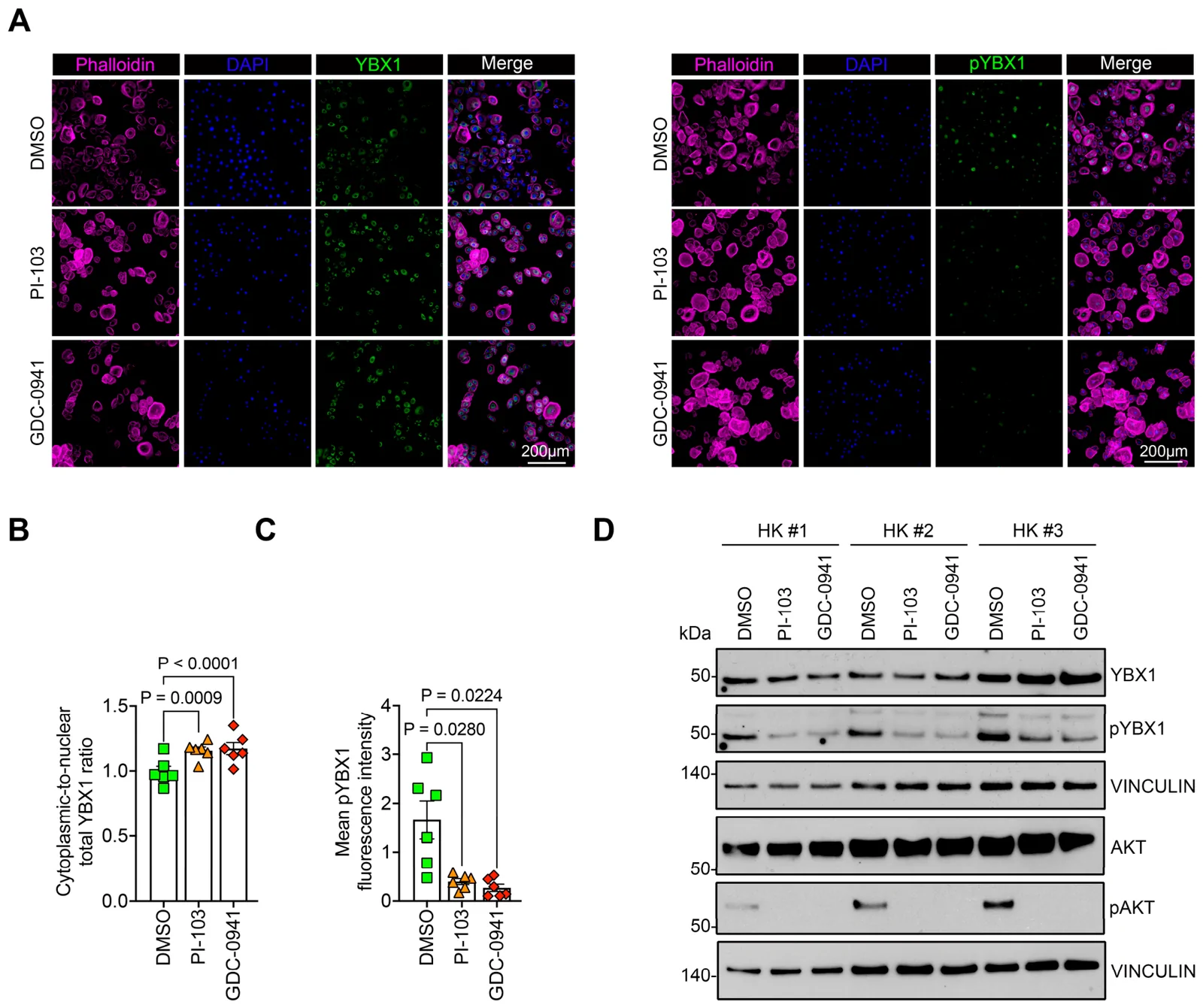
PI3K/AKT pathway inhibition reduces YBX1 phosphorylation across independent human keratinocyte donors. (**A**) Representative immunofluorescence images of total YBX1 (**left panel**, green) and pYBX1 (**right panel**, green) in primary human keratinocytes (HK) treated with DMSO, 1 μM PI-103, or 1 μM GDC-0941 for 2 h. DAPI (blue) and phalloidin (magenta) were used as nuclear and cytoplasmic markers, respectively. Scale bar, 200 μm. (**B**,**C**) Quantification of the cytoplasmic/nuclear YBX1 ratio (**B**) and pYBX1 (**C**) fluorescence intensity. n = 6 donors. (**D**) Western blot analysis of YBX1, pYBX1, AKT, and pAKT in HK treated as in (**A**). Vinculin was used as a loading control. All values are shown as mean ± SEM; one-way ANOVA followed by Dunnett’s multiple comparisons test for (**B**,**C**).

To confirm that this selective effect on YBX1 phosphorylation was not restricted to immortalized cell lines and held across individuals, we extended the analysis to primary human keratinocytes (HK) from independent donors. Immunofluorescence analysis showed that treatment with PI-103 or GDC-0941 significantly decreased nuclear pYBX1 staining and increased the cytoplasmic-to-nuclear ratio of total YBX1, indicating redistribution of YBX1 toward the cytoplasm. (Figure 3A–C). Consistent with the results obtained in Ker-CT, immunoblotting in three independent HK donors confirmed the loss of pAKT and reduced pYBX1 alongside preserved total YBX1 after treatment with PI-103 or GDC-0941 (Figure 3D). These effects occurred despite only modest, dose-dependent reductions in HK viability over the range tested (Supplementary Figure S3A). The suppression of YBX1 phosphorylation by PI3K/AKT pathway inhibition was therefore reproducible across donors in primary human keratinocytes, prompting us to ask whether this translates into a change in the senescent phenotype.

### 3.4. Inhibition of YBX1 Phosphorylation Attenuates SASP Chemokine Translation and Secretion Reducing Keratinocyte Senescence

**Figure 4:**
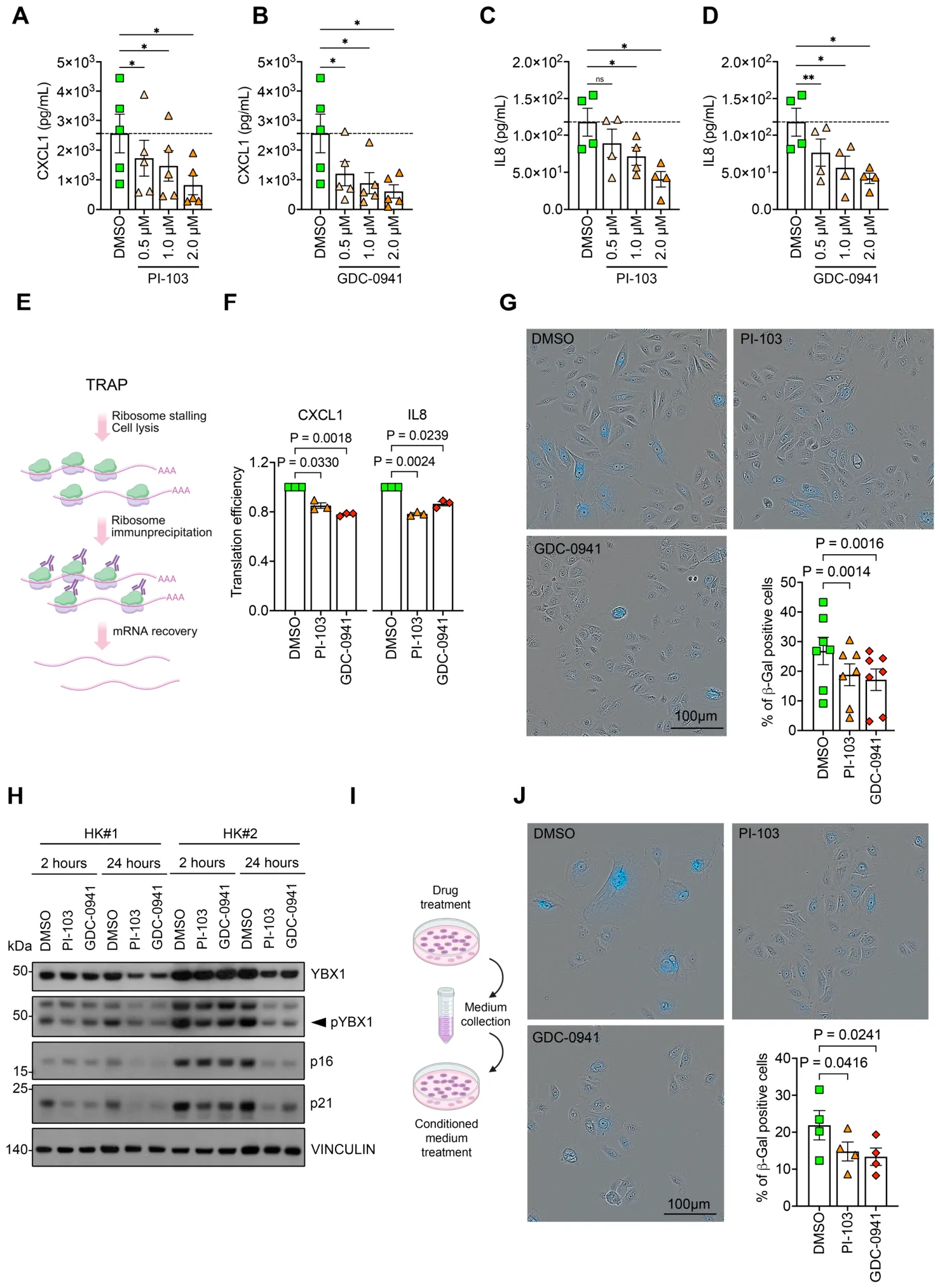
Inhibition of YBX1 phosphorylation attenuates SASP chemokine translation and secretion and keratinocyte senescence. (**A**,**B**) Quantification of CXCL1 secretion by ELISA in supernatants of HK treated with DMSO or increasing concentrations of PI-103 (**A**) or GDC-0941 (**B**) for 48 h. n = 5 donors. (**C**,**D**) Quantification of IL8 secretion by ELISA in supernatants of HK treated with DMSO or increasing concentrations of PI-103 (**C**) or GDC-0941 (**D**) for 48 h. n = 4 donors. * = *p* < 0.05, ** = *p* < 0.01, ns = not significant. (**E**) Schematic of Translating Ribosome Affinity Purification (TRAP). (**F**) Translational efficiency (TE) of CXCL1 and IL8, calculated as the ratio of ribosome-associated to total mRNA, in HK following treatment with DMSO, 1 μM PI-103, or 1 μM GDC-0941 for 2 h, determined by TRAP. n = 3 donors. (**G**) Representative images of SA-β-Gal staining in HK treated with DMSO, 1 µM PI-103, or 1 µM GDC-0941 for 72 h, and quantification of the proportion of SA-β-Gal-positive cells. Scale bar, 100 μm. n = 7 donors. (**H**) Western blot analysis of YBX1, pYBX1, p16^INK4a^, and p21^CIP1^ in HK treated with DMSO, 1 µM PI-103, or 1 µM GDC-0941 for 24 h. Vinculin was used as a loading control. (**I**) Schematic of the conditioned medium transfer experiment: HK were treated with DMSO, 1 μM PI-103, or 1 μM GDC-0941 for 24 h. The medium was then replaced, and cells were incubated for an additional 24 h before the conditioned medium was collected and transferred to untreated HK. (**J**) Representative images of SA-β-Gal staining in recipient HK after 48 h of incubation with the conditioned medium, and quantification of the proportion of SA-β-Gal-positive cells. Scale bar, 100 μm. n = 4 donors. All values are shown as mean ± SEM; one-way ANOVA followed by Dunnett’s multiple comparisons test for (**A**–**D**,**F**,**G**,**J**). The schematics in (**E**,**I**) were created using BioRender.

Having established that PI3K/AKT pathway inhibition suppresses YBX1 phosphorylation, we asked whether this restrains SASP-associated cytokine secretion and the senescent phenotype of keratinocytes. Both PI-103 and GDC-0941 reduced CXCL1 secretion in a dose-dependent manner (Figure 4A,B), and similarly lowered IL8 secretion, with GDC-0941 reaching significance at the lowest dose tested (Figure 4C,D). To determine whether this reflected translational control, as predicted by the repression of the *CXCL1* and *IL8* 3′UTRs by YBX1, we measured ribosome-associated transcript by ribosome immunoprecipitation followed by mRNA recovery (Figure 4E). We then determined the translational efficiency of *CXCL1* and *IL8* by comparing ribosome-associated transcript levels with their corresponding total mRNA abundance. Both inhibitors significantly decreased the translational efficiency of *CXCL1* and *IL8*, indicating that reduced translation, rather than reduced transcript abundance, was responsible for the drop in secreted chemokine (Figure 4F). Mechanistically, the loss of pYBX1, at constant total YBX1, enriches the unphosphorylated form available to bind the *CXCL1* and *IL8* 3′UTRs, thereby reinforcing translational repression of these transcripts and lowering their secretion.

To determine if reduced SASP chemokine translation was accompanied by attenuation of the senescent phenotype, we performed a β-galactosidase senescence assay in human keratinocytes treated with PI-103 or GDC-0941. After 72 h of treatment, both inhibitors significantly decreased the proportion of β-galactosidase (SA-β-Gal)-positive keratinocytes compared with the vehicle control (Figure 4G), suggesting that reduced YBX1 phosphorylation is associated with attenuation of the senescent phenotype. Consistent with the reduction in SA-β-Gal-positive cells, both PI-103 and GDC-0941 decreased p16^INK4a^ and p21^CIP1^ protein levels relative to DMSO-treated controls, concomitant with reduced pYBX1 levels while preserving total YBX1 expression (Figure 4H). Because CXCL1 and IL8 reinforce senescence in a paracrine manner [11,12], we tested whether this effect was mediated through secreted factors. Conditioned medium collected from PI-103- or GDC-0941-treated cells reduced SA-β-Gal staining in untreated recipient keratinocytes compared with conditioned medium from DMSO-treated cells (Figure 4I,J). Together, these results indicate that PI3K/AKT pathway inhibition reduces SASP chemokine secretion and attenuates keratinocyte senescence, consistent with a mechanism involving reduced pYBX1 and restoration of YBX1-mediated translational repression of *CXCL1* and *IL8* through both direct and paracrine mechanisms.

### 3.5. YBX1 Is Required for the Anti-Senescence Effect of PI3K/AKT Pathway Inhibition

**Figure 5:**
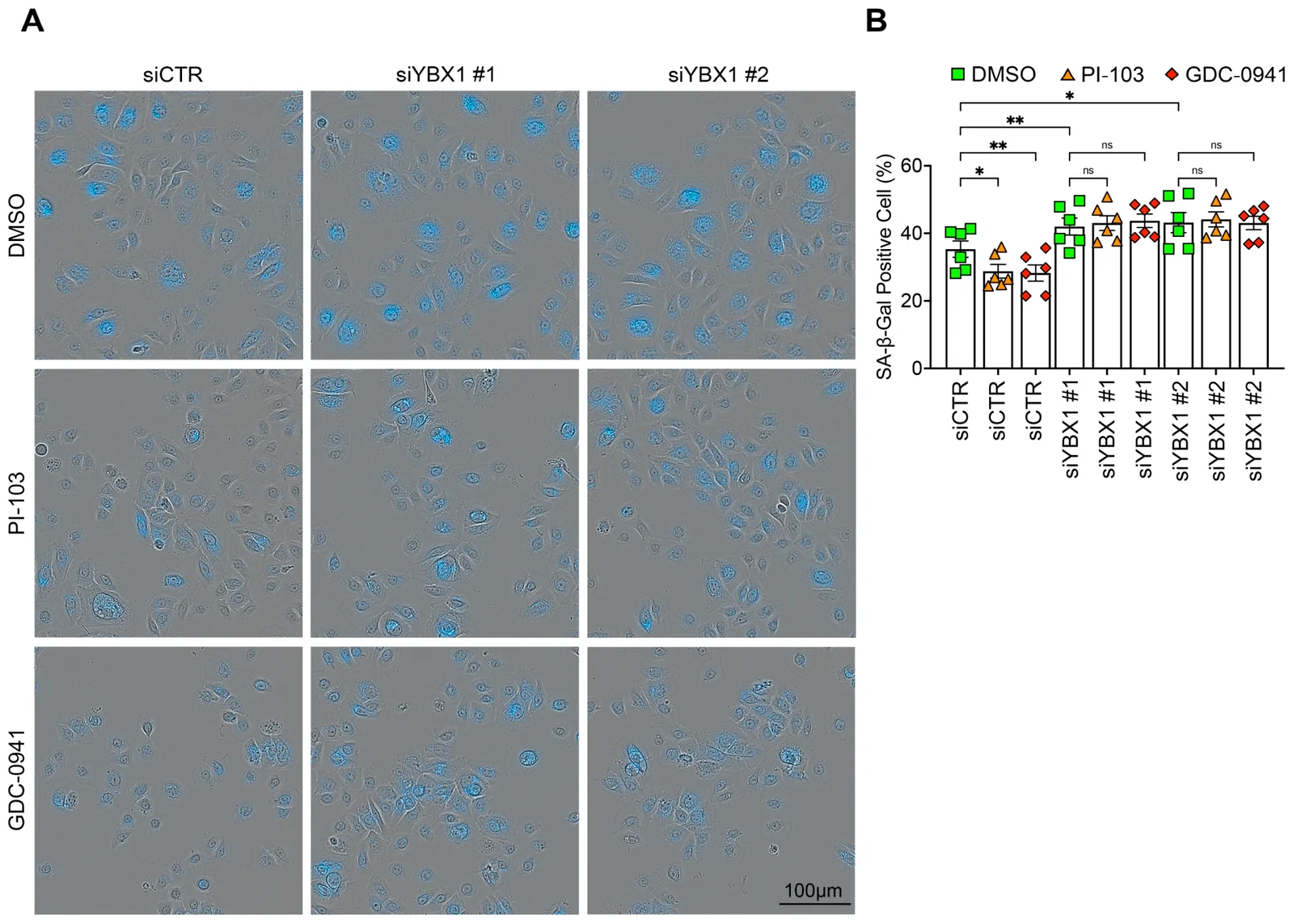
YBX1 is required for the anti-senescence effect of PI3K/AKT pathway inhibition. (**A**,**B**) Representative images of SA-β-Gal staining in primary human keratinocytes (HK) transfected with control siRNA (siCTR) or YBX1-targeting siRNA (siYBX1 #1 or #2) and subsequently treated with DMSO, 1 µM PI-103, or 1 µM GDC-0941 for 72 h (**A**) and quantification of the proportion of SA-β-Gal-positive cells (**B**). Scale bar, 100 μm. n = 6 biological replicates. * = *p* < 0.05, ** = *p* < 0.01, ns = not significant. All values are shown as mean ± SEM; one-way ANOVA followed by Dunnett’s multiple comparisons test for (**B**).

The preceding experiments established that PI3K/AKT inhibition attenuates keratinocyte senescence but did not determine whether this effect requires YBX1. To address this directly, we silenced YBX1 (Supplementary Figure S4A) and treated cells with PI-103 or GDC-0941, followed by assessment of SA-β-Gal. In control cells, both inhibitors reduced the proportion of SA-β-Gal-positive keratinocytes, consistent with our previous findings. YBX1 depletion increased baseline senescence, consistent with loss of its repressive function, and abolished the ability of either inhibitor to reduce SA-β-Gal positivity (Figure 5A,B). This epistasis, loss of the inhibitor effect upon YBX1 removal, indicates that the anti-senescence effect of PI3K/AKT inhibition is YBX1-dependent. Thus, YBX1 is required for the anti-senescence effect of PI3K/AKT inhibition: in its absence, neither inhibitor was able to reduce senescence, demonstrating that this effect is mediated, at least in part, through YBX1 rather than through other targets of the pathway.

### 3.6. Direct Inhibition of YBX1-Phosphorylating Kinases Recapitulates PI3K/AKT Pathway Inhibitor Effects and Reduces Keratinocyte Senescence

Although PI-103 and GDC-0941 effectively suppressed YBX1 phosphorylation, the PI3K/AKT pathway regulates numerous cellular processes beyond YBX1, including cell growth, proliferation, survival, and metabolism [13] and, in the context of skin, is required to maintain epidermal barrier function [14]. Broad inhibition of this upstream pathway may therefore carry a risk of off-target effects that could exacerbate features of aging skin. We therefore sought to determine whether the observed phenotype could be reproduced by selectively targeting kinases responsible for YBX1 phosphorylation. Although AKT is the best-characterized kinase regulating YBX1 Ser102 phosphorylation, p90^RSK^ has also been reported to phosphorylate the same residue. Thus, we tested the effects of the selective AKT inhibitor MK-2206 and the p90^RSK^ inhibitor SL0101 in Ker-CT cells. Cell viability was first assessed to determine the optimal concentration of each drug for subsequent experiments (Supplementary Figure S5A). Based on these results, 25 and 50 μM SL0101 and 5 and 10 μM MK-2206 were selected for subsequent experiments.

**Figure 6:**
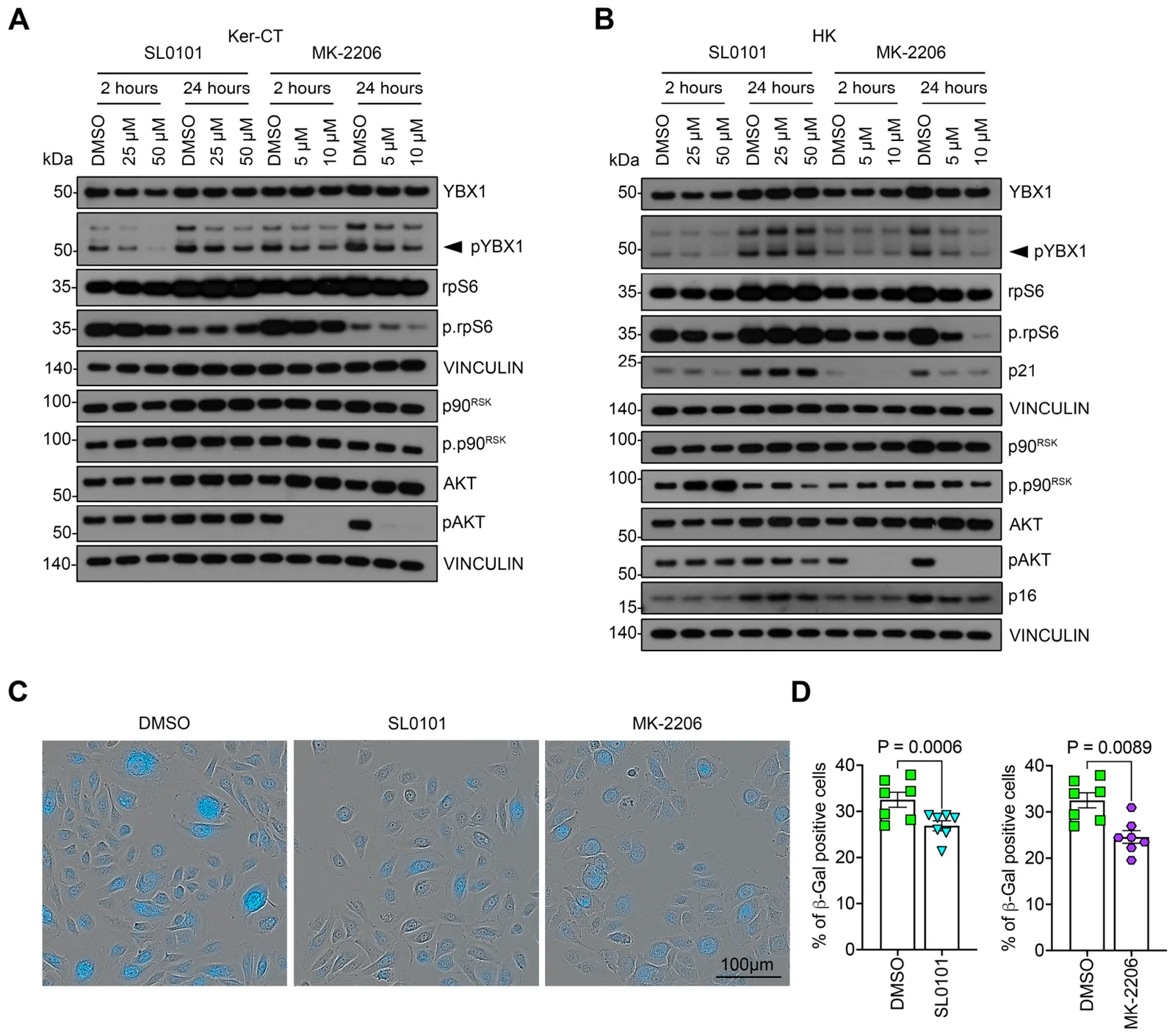
Direct inhibition of YBX1-phosphorylating kinases recapitulates the effects of PI3K/AKT pathway inhibitors. (**A**) Western blot analysis of YBX1, pYBX1, p-rpS6, rpS6, p90^RSK^, phospho-p90^RSK^, AKT and pAKT in Ker-CT cells treated with the indicated concentrations of SL0101 or MK-2206 for 2 or 24 h. Vinculin was used as a loading control. (**B**) Western blot analysis of YBX1, pYBX1, p-rpS6, rpS6, p90^RSK^, phospho-p90^RSK^, AKT pAKT, p16^INK4a^, and p21^CIP1^ in HK cells treated with the indicated concentrations of SL0101 or MK-2206 for 2 or 24 h. Vinculin was used as a loading control. (**C**,**D**) Representative images of SA-β-Gal staining in HK treated with DMSO, 50 µM SL0101, or 10 µM MK-2206 for 72 h (**C**) and quantification of the proportion of SA-β-Gal-positive cells (**D**). Scale bar, 100 μm. n = 7 donors (SL0101) and n = 7 donors (MK-2206). All values are shown as mean ± SEM; Welch’s two-tailed *t*-test was performed for (**D**).

Both inhibitors reduced YBX1 phosphorylation in a dose-dependent manner (Figure 6A), comparable to the effects observed with PI-103 and GDC-0941. As expected, treatment with MK-2206 resulted in loss of pAKT, confirming effective AKT inhibition. In the case of SL0101, the phosphorylation of p90^RSK^ itself was unchanged, consistent with its mechanism of action: SL0101 is an ATP-competitive inhibitor that occupies the p90^RSK^ kinase domain and blocks its catalytic activity without preventing phosphorylation of the kinase. To monitor p90^RSK^ inhibition functionally, we therefore assessed the phosphorylation of ribosomal protein S6 (p-rpS6), a downstream RSK substrate; p-rpS6 was reduced by SL0101, confirming effective inhibition of RSK signaling. Notably, SL0101 suppressed YBX1 and rpS6 phosphorylation most strongly at 2 h, with a loss of effect by 24 h. This time-dependence is consistent with the reported low biological stability of SL0101, whose acetyl groups are susceptible to esterase-mediated cleavage and whose short half-life limits sustained target engagement [15,16].

These findings were recapitulated in primary human keratinocytes, with both MK-2206 and SL0101 reducing YBX1 phosphorylation, lowering the senescence markers p16^INK4a^ and p21^CIP1^ (Figure 6B), and decreasing the proportion of SA-β-Gal-positive cells relative to DMSO-treated controls (Figure 6C,D).

Together, these results demonstrate that direct pharmacologic inhibition of the kinases that phosphorylate YBX1 is sufficient to recapitulate the effects of upstream PI3K/AKT inhibition on keratinocyte senescence. By bypassing the pleiotropic effects of the PI3K/AKT upstream pathway inhibition, these findings position AKT- and p90^RSK^-directed inhibition of YBX1 phosphorylation as a compelling candidate strategy for future studies aimed at selectively modulating the keratinocyte SASP and mitigating intrinsic skin aging.

## 4. Discussion

Skin aging is accompanied by the accumulation of senescent keratinocytes whose secretome sustains a chronic inflammatory environment [5,17], yet the upstream regulators that gate SASP production in the epidermis remain incompletely defined. While previous studies have established YBX1 as a post-transcriptional regulator of inflammatory cytokine translation, how its activity is regulated during physiological aging has remained unknown [6]. Here, we identify YBX1 phosphorylation as an age-associated molecular switch that controls the translational output of the keratinocyte SASP. Rather than a simple reduction in YBX1 abundance, physiological skin aging is characterized by a shift in the phosphorylation state of the remaining YBX1 pool, thereby reducing the cytoplasmic fraction available to repress translation of SASP-associated chemokine mRNAs.

Our central observation is a dissociation between YBX1 abundance and YBX1 activation. Total YBX1 declines in aged epidermis, consistent with the loss of cycling basal progenitors in which the protein is enriched, whereas pYBX1 is preserved. The resulting rise in the pYBX1/total YBX1 ratio, which increases with donor chronological age, indicates that aged epidermis does not simply lose YBX1 but shifts the residual pool toward a phosphorylated state. This distinction is mechanistically decisive because it is the unphosphorylated, cytoplasmic form of YBX1 that engages target 3′UTRs and represses their translation [18,19]. It follows that the pYBX1/total YBX1 ratio, rather than total YBX1 protein, is the more informative readout of YBX1 activity in aged tissue.

We tested this model pharmacologically. The PI3K inhibitors PI-103 and GDC-0941 lowered pAKT and pYBX1 without depleting total YBX1 and repressed reporter expression driven by the *CXCL1* and *IL8* 3′UTRs in a dose-dependent manner. This effect was reproducible across independent primary human keratinocyte donors, arguing against a peculiarity of the immortalized Ker-CT line. The selective loss of pYBX1 at constant total YBX1 necessarily enriches the unphosphorylated cytoplasmic form, which can bind the *CXCL1* and *IL8* 3′UTRs, repressing their translation. Indeed, ribosome immunoprecipitation showed that both inhibitors reduced the translational efficiency of *CXCL1* and *IL8*, placing the regulation at the level of translation rather than transcript abundance and confirming, in the context of aging, the post-transcriptional mechanism we originally described in epidermal progenitors [6]. Reduced translation was accompanied by lower secretion of both chemokines and a decline in SA-β-Gal-positive keratinocytes.

The conditioned-medium experiment further supports the involvement of paracrine signaling in this process. Medium from inhibitor-treated cells reduced senescence in untreated recipient keratinocytes, indicating that dampening the secretome is by itself sufficient to attenuate senescence in neighboring cells. This is consistent with the established capacity of CXCL1 and IL8 to reinforce and propagate the senescent state in a paracrine manner [12,20] and positions the YBX1-controlled chemokine output as a node through which senescence spreads within the epidermis rather than merely a marker of it.

A translational concern arises from the extent of PI3K/AKT signaling, which governs keratinocyte growth, survival, metabolism, and epidermal barrier integrity [14]. Sustained pathway blockade would plausibly exacerbate, rather than mitigate, the epidermal atrophy that characterizes aged skin. Our observation that the direct kinase inhibitors MK-2206 (AKT) and SL0101 (p90^RSK^) recapitulate the reduction in pYBX1 and in SA-β-Gal–positive cells are therefore significant: it indicates that the senescence phenotype can be uncoupled from broad upstream pathway inhibition. The comparable efficacy of AKT- and p90^RSK^-directed inhibition further suggests that YBX1 phosphorylation in keratinocytes is regulated by two convergent kinases [21], consistent with YBX1 acting as an integrator of growth-factor and stress signaling. Whether combined or selective targeting offers a wider therapeutic window remains to be determined.

Several limitations should be acknowledged. Although the concordance between pharmacological inhibition, altered YBX1 localization, reduced translational efficiency of CXCL1 and IL8, diminished cytokine secretion, and attenuation of senescence supports a role for YBX1 phosphorylation in the regulation of senescence-associated phenotype, definitive demonstration of causality will require genetic manipulation of the Ser102 phosphorylation site or phospho-mutant rescue experiments. In addition, our analyses were performed using cultured primary human keratinocytes and a cross-sectional cohort of human abdominal skin. Future studies using genetically engineered models and topical pharmacological approaches will be necessary to determine whether modulation of YBX1 phosphorylation can safely attenuate skin aging in vivo while preserving normal epidermal homeostasis.

Despite these considerations, our findings converge on a coherent model in which age-associated phosphorylation of YBX1 lifts a physiological restraint on SASP chemokine translation, and in which restoring that restraint, by inhibiting the kinases responsible, suppresses the secretory phenotype and is associated with reduced paracrine propagation of senescence. Because senomorphic strategies aim to mitigate the deleterious secretome without eliminating senescent cells [22], the YBX1 phosphorylation switch may represent an attractive point of intervention: it acts post-transcriptionally on a defined set of transcripts, and it is accessible through existing kinase inhibitors.

## 5. Conclusions

In conclusion, our findings identify YBX1 phosphorylation as an age-associated translational switch controlling the keratinocyte SASP. By regulating the balance between cytoplasmic translational repression and nuclear signaling, phosphorylation of YBX1 determines inflammatory chemokine production and may thereby contribute to the propagation of cellular senescence within the epidermis. These results expand our understanding of the molecular mechanisms underlying intrinsic skin aging and establish post-transcriptional regulation of inflammatory translation as a previously underappreciated therapeutic target for senomorphic intervention.

## Figures and Tables

**Table 1:** List of primer pairs used for RT-qPCR analysis.

Gene	Forward Primer (5′–3′)	Reverse Primer (5′–3′)
*RPLP0*	TGGTCATCCAGCAGGTGTTCGA	ACAGACACTGGCAACATTGCGG
*CXCL1*	AGCTTGCCTCAATCCTGCATCC	TCCTTCAGGAACAGCCACCAGT
*IL8*	AAGTTTCACTGGCATCTTCACTG	GAGAAGTTTTTGAAGAGGGCTGAG

## Data Availability

Correspondence and requests for materials should be addressed to Stefano Sol and Anna Mandinova.

## Supplementary Materials

The following supporting information can be downloaded at: https://www.mdpi.com/article/10.3390/cells15171586/s1, Figure S1: Old human skin samples display markers of chronological aging; Figure S2: The effect of PI3K/AKT pathway inhibition on YBX1 phosphorylation is recapitulated in OKF6-TERT2 keratinocytes; Figure S3: Effect of PI3K inhibitors on primary human keratinocyte viability; Figure S4: Validation of YBX1 silencing in primary human keratinocytes; Figure S5: Effect of PI3K/AKT pathway inhibitors on Ker-CT.

## Abbreviations

YBX1	Y-box binding protein 1
pYBX1	Phosphorylated Y-box binding protein 1
HK	Human keratinocyte
SASP	Senescence-associated secretory phenotype
SA-β-Gal	Senescence-Associated β-Galactosidase
TRAP	Translating Ribosome Affinity Purification
