# Supplementary material for: Age-Associated YBX1 Phosphorylation Regulates the Keratinocyte Senescence-Associated Secretory Phenotype Through Translational Control
Source: Cells. 2026 Sep 1;15(17):1586. doi: 10.3390/cells15171586 (PMC13564713; doi:10.3390/cells15171586)
Supplement: Supplementary file 1 [file cells-15-01586-s001.zip › cells-4485941-supplementary.pdf]

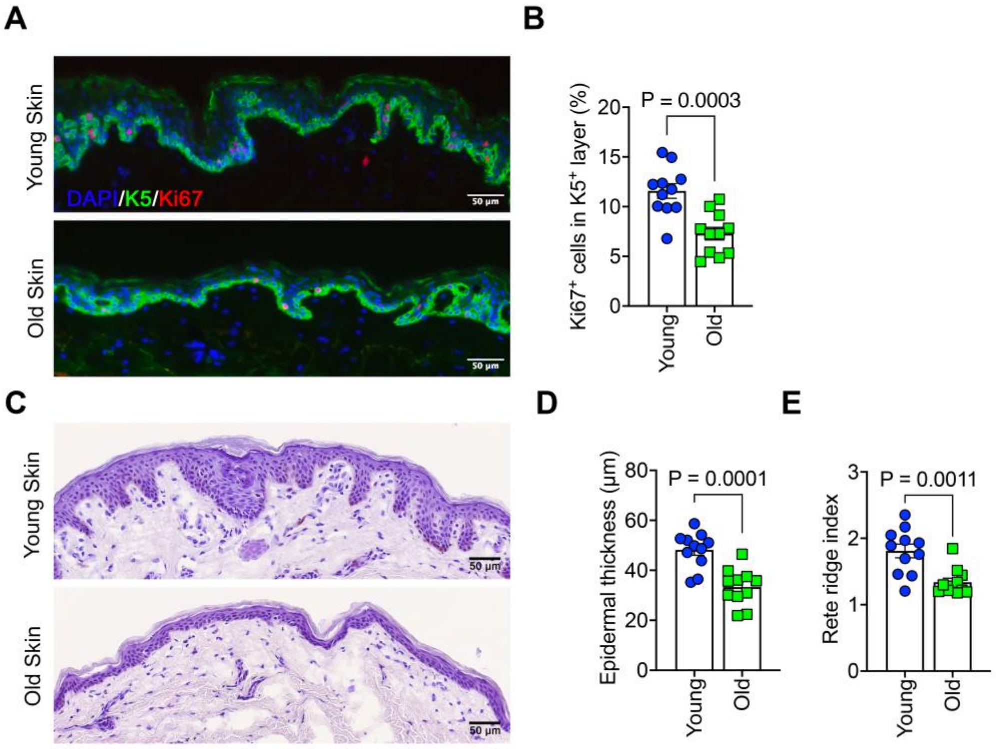

Figure S1. Old human skin samples display markers of chronological aging. (**A**, **B**) Representative immunofluorescence images of Ki-67 (red) in young (upper panel) and old (lower panel) human skin samples (**A**) and quantification of Ki-67+ cells within the K5+ layer (**B**). DAPI (blue) and K5 (green) were used as nuclear and basal-layer markers, respectively. Scale bar, 50 µm. (**C-E**) Representative H&E staining of young (upper panel) and old (lower panel) human skin (**C**) and quantification of epidermal thickness (**D**) and rete ridge index (**E**). Epidermal thickness was measured as the perpendicular distance from the dermal papilla to the stratum corneum; rete ridge index was calculated as the ratio of the actual length of the dermal-epidermal junction to the horizontal length of the measured skin section. n = 11 young donors and n = 11 old donors. All values are shown as mean ± SEM; Welch's two-tailed t-test was performed for **B**, **D**, and **E**.

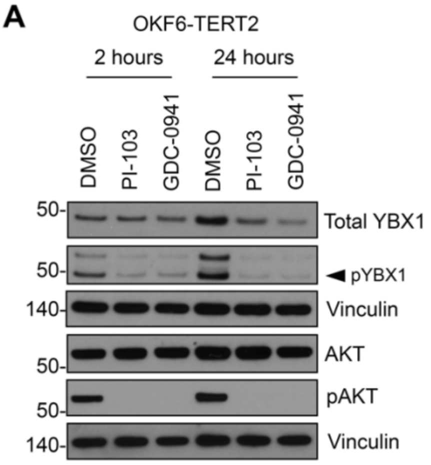

Figure S2. The effect of PI3K/AKT pathway inhibition on YBX1 phosphorylation is recapitulated in OKF6-TERT2 keratinocytes. (**A**) Western blot analysis of YBX1, pYBX1, AKT, and pAKT in

OKF6-TERT2 cells treated with DMSO, 1 μM PI-103, or 1 μM GDC-0941 for 2 or 24 hours. Vinculin was used as a loading control.

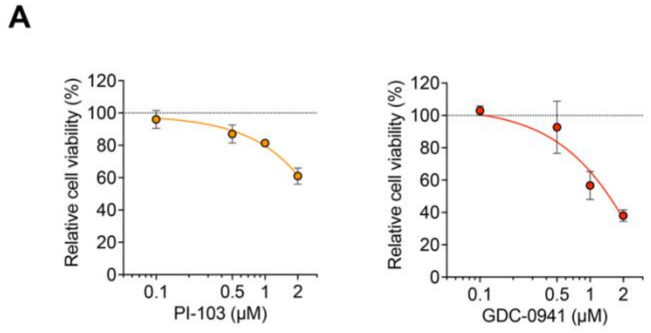

Figure S3. Effect of PI3K inhibitors on primary human keratinocyte viability. (**A**) MTT assay in HK after 48 hours of treatment with the indicated concentrations of DMSO, PI-103 (left panel), or GDC-0941 (right panel). Relative cell viability was normalized to DMSO. n = 3 donors. All values are shown as mean ± SEM.

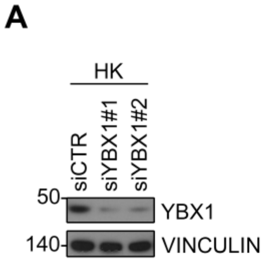

Figure S4. Validation of YBX1 silencing in primary human keratinocytes. (**A**) Western blot analysis of YBX1 in primary keratinocytes transfected with control siRNA (siCTR) or YBX1-targeting siRNA (siYBX1 #1 or #2). Vinculin was used as a loading control.

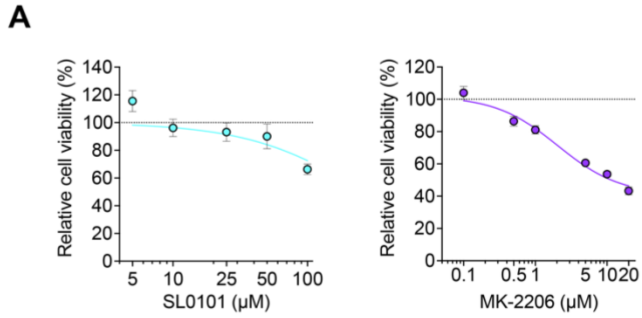

Figure S5. Effect of PI3K/AKT pathway inhibitors on Ker-CT. (**A**) MTT assay in KerCT after 48 hours of treatment with the indicated concentrations of DMSO, SL0101 (left panel), or MK-2206 (right panel). Relative cell viability was normalized to DMSO. n = 4 biological replicates. All values are shown as mean ± SEM.
